# Effects of delayed cord clamping on neonatal jaundice, phototherapy and early hematological status in term cesarean section

**DOI:** 10.1186/s13052-021-01069-6

**Published:** 2021-05-26

**Authors:** Hailing Shao, Shichu Gao, Qiujing Lu, Xiaomin Zhao, Ying Hua, Xiaomei Wang

**Affiliations:** 1 Department of Obstetrics and Gynecology, the Second Affiliated Hospital of Wenzhou Medical University, Wenzhou, 325027 China; 2 Department of Obstetrics and Gynecology, Jiaxing Xiuzhou District Maternal and Child Health Hospital, Zhejiang, China

**Keywords:** Delayed cord clamping, Neonatal jaundice, Phototherapy, Anemia, Cesarean section

## Abstract

**Background:**

Delayed cord clamping in full-term neonates is widely recommended, while in practice, it is rarely implemented in cesarean section due to the fear of neonatal jaundice and excessive maternal blood loss. The optimal timing of cord clamping remains uncertain. This study was to fully evaluate the effects of delayed cord clamping on short-term hematological status and jaundice in term neonates delivered by cesarean section.

**Methods:**

This retrospective study enrolled 796 women, who were allocated into the early cord clamping group (*n* = 377) and the delayed cord clamping group (*n* = 419). The latter group was further divided into two subgroups (30–60 s, 61–120 s).

The outcomes were neonatal transcutaneous bilirubin levels on 0 to 5 days of life and the rate of phototherapy. For neonates who had blood tests on the first three days of life, their hemoglobin and hematocrit were compared among groups.

**Results:**

Compared with the early cord clamping group, delayed cord clamping merely increased the transcutaneous bilirubin level of neonates on the day of birth rather than that on the following five days. The heel peripheral blood sample size of 1–3 days in the early cord clamping group was 61, 25 and 33, and in the delayed cord clamping group was 53, 46 and 32, respectively. Delayed cord clamping at 30–60 s resulted in the higher neonatal hemoglobin level on day 3 and an increased rate of neonatal polycythemia, without a higher rate of phototherapy. Delayed cord clamping beyond 60 s did not further improve hematological status in term neonates born by cesarean section.

**Conclusion:**

In cesarean section, delayed cord clamping for 30–60 s improved the early hematological status of term neonates without the enhanced requirement of phototherapy for neonatal jaundice.

## Background

Over the past decade, the American College of Obstetricians and Gynecologists (ACOG) had changed the recommendation of delayed cord clamping (DCC) at least 30–60 s after birth from for preterm neonates only [1] to for both preterm and vigorous term neonates [2], under a well-established monitoring and treatment system for neonatal jaundice.

Hyperbilirubinemia is considered a potential disadvantage of DCC, while in practice, it does not appear to be associated with increased phototherapeutic demand [3–5]. As a simple and safe procedure, DCC proves to be beneficial for a better hematological status in the first several hours or months of full-term newborn’s life, including hemoglobin, hematocrit levels [3, 6–8], and iron status [9]. Iron deficiency anemia in neonates is a worldwide concerned health issue because of its relationship with poorer cognitive, motor, auditory and social-emotional function [10–12]. Thus, the implementation of DCC should be advocated.

Most randomized controlled trials focused on the population of neonates delivered vaginally. However, the pattern of placental transfusion in vaginal delivery is different from that in cesarean section. In the 1960s, Yao et al. demonstrated that the blood volume of full-term neonates born through vagina increased by 19.3% at 1-min-delayed cord clamping and 32% when umbilical cord pulsation ceased [13]. Under the condition of cesarean section, clamping the cord beyond 40 s reversed the net flow between the placenta and neonate, resulting in a rebound of the residual placental blood volume [14]. A systematic review and meta-analysis revealed cesarean section was related to a less placental transfusion compared with vaginal delivery [15].

It is reasonable that the role of DCC in the two delivery methods is inconsistent, and this topic is of great clinical significance. Several studies focused on the effect of DCC on early neonatal hematological status in neonates born to the mothers who underwent cesarean section, but the results were inconsistent [7, 16, 17]. Moreover, these studies only examined neonatal bilirubin at a single point after birth. In this study, to investigate the benefits of DCC and its optimal timing in term cesarean section, we fully assessed the effects of DCC (30–60 s, 61–120 s) in term neonates with cesarean section, including transcutaneous bilirubin levels on day 0 to day 5 after birth, the rate of phototherapy and hemoglobin and hematocrit levels of neonates on the first three days.

## Methods

This retrospective trial was conducted by reviewing the electronic medical records of Jiaxing Xiuzhou District Maternal and Child Health Hospital in Province Zhejiang in China from April 1st, 2018 to April 30th, 2019. When admitted to the hospital for delivery, all participants signed informed consent that they agreed to the instructions of doctors and using their clinical data for scientific research. This study obtained the ethical approval of Jiaxing Xiuzhou District Maternal and Child Health Hospital for Women & Newborns Human Research Protection Office.

Eligible participants should meet all the following criteria: (1) singleton pregnancy; (2) term pregnancy with the gestational age of 37–42 weeks; (3) delivered by elective cesarean section; (4) mothers: Rhesus D-positive blood; normal pregnancy without hypertension disorders, diabetes mellitus, intrahepatic cholestasis of pregnancy, polyhydramnios, oligohydramnios, placenta previa, and placental abruption; (5) neonates: birth weight of 2500–4000 g; no resuscitation at birth; healthy neonates without congenital malformations (anal atresia, biliary atresia, heart disease), pneumonia, and any other diseases influencing serum bilirubin levels; (6) Women received cord clamping less than 15 s or more than 30 s after the delivery of neonates.

This study included the early cord clamping (ECC) group, the 30–60 s DCC subgroup and the 61–120 s subgroup. We assumed that the group allocation ratio was 2:1:1 and transcutaneous bilirubin on the day of birth was the main outcome. Sample sizes of 314, 157, and 157 were obtained from the 3 groups by PASS software. We reviewed the timing of umbilical cord clamping of eligible participants in the electronic medical record database from April 1st, 2018 to April 30th, 2019. Women enrolled were respectively allocated into the ECC group with the umbilicus clamped less than 15 s or the DCC group with the umbilicus clamped beyond 30 s. Further, the DCC group was divided into two subgroups according to the timing of cord clamping (30–60 s, 61–120 s). Surgeries were performed by obstetricians who had the same operative technique. After birth, neonates were placed between the legs of mothers where the umbilical cord was kept free of tension.

Baseline characteristics were recorded, such as age, gestational age, fetal birth weight, and Apgar scores at 1 min and 5 min. Heel peripheral blood samples were collected by trained nurses on day 1, 2 and 3, from which the hemoglobin and hematocrit were tested. This procedure was decided by neonatologists for other medical considerations, not for this study. The transcutaneous bilirubin on day 0 to day 5 after birth was measured using the uniform TcB device three times a day (JM-103, KONICA MINOLTA, Japan) and the highest value was recorded. The neonatal attending physician decided to implement phototherapy when the neonates were considered hyperbilirubinemia based on transcutaneous and serum bilirubin levels. Polycythemia is defined as a hematocrit value > 65%. The number of neonates with polycythemia in each group was recorded.

Statistical analysis was performed by SPSS 25.0 software. Continuous variables were presented as mean ± standard deviation (SD) or median and interquartile range (IQR). Normally distributed data were analyzed by Student t-test or one-way ANOVA with LSD post hoc, and non-normally distributed data by Mann-Whitney U test between the ECC group and the ECC group. Categorical variables, presented as percentage (%), including the rate of phototherapy and polycythemia, were analyzed by Pearson’s Chi-square test or Fisher exact probability test. All *P*-values were two-sided and if below 0.05 the results were considered statistically significant.

## Results

**Fig. 1:**
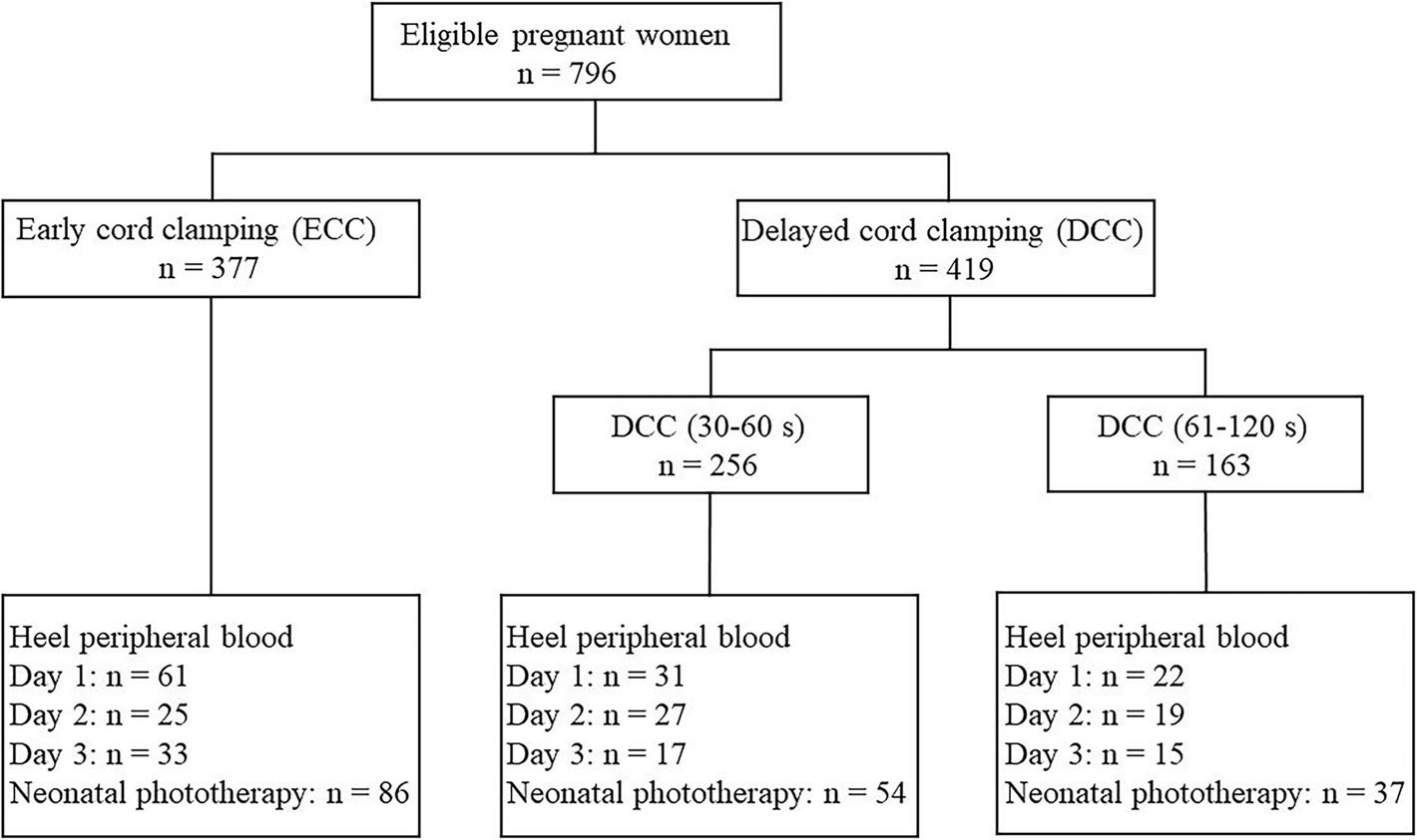
Study flow chart

As is shown in Figs. 1, 796 women were enrolled in our study, of which the distribution is 377 cases in the ECC group and 419 cases in the DCC group. DCC group was divided into the 30–60 s DCC group (*n* = 256) and the 61–120 s DCC group (*n* = 163). Not all neonates had hematological results. The heel blood sample size of 1–3 days in the ECC group was 61, 25 and 33, and in the DCC group was 53, 46 and 32, respectively (Fig. 1).

There were no significant differences in age, gravidity, parity, gestational age, fetal birth weight, fetal sex and Apgar score at 1 min and 5 min between the ECC group and DCC group or DCC subgroups (*p* > 0.05) (Table 1). The median time of cord clamping was less than 15 s (data not shown) in the ECC group, 60 s (IQR 50–70) in the DCC group, 53.5 s (IQR 45–60) in the 30–60 s DCC group, and 76 s (IQR 70–86) in the 61–120 s DCC group. The results of neonatal transcutaneous bilirubin on day 0 to day 5 after birth were given in Table 2. On day 0, the transcutaneous bilirubin in the DCC group (1.70 ± 0.78 mg/dl) and the 30–60 s DCC group (1.73 ± 0.80 mg/dl) was markedly higher than that in the ECC group (1.49 ± 0.90 mg/dl) (*p* < 0.05). Though there was a slightly increased transcutaneous bilirubin level in the 61–120 s DCC group (1.66 ± 0.74 mg/dl) compared with the ECC group, it was not statistically significant (*p* > 0.05). However, no differences were found in the following 5 days among any groups (*p* > 0.05). The rate of phototherapy was 22.8% (86/377), 21.1% (54/256) and 22.7% (37/163) in the ECC, DCC (30–60 s) and DCC (61–120 s) group, respectively. Either clamping the cord at 30–60s or beyond 60 s did not increase the requirement of phototherapy for neonatal jaundice compared with the ECC group (*p* > 0.05).

**Table 1:** Baseline characteristics of the study groups

	ECC group (*n* = 377)	DCC group (*n* = 419)	DCC (30–60 s) group (*n* = 256)	DCC (61–120 s) group (*n* = 163)	*p*^a^	*p*^b^
Mother’s age (years)	28.99 ± 4.58	28.99 ± 4.27	29.11 ± 4.27	28.79 ± 4.26	0.997	0.773
Gravidity	3 (2, 3)	3 (2, 4)	3 (2, 4)	3 (2, 4)	0.983	0.986
Parity	2 (2, 2)	2 (2, 2)	2 (2, 2)	2 (2, 2)	0.314	0.426
Gestational age (weeks)	38.90 ± 0.79	38.91 ± 0.78	38.90 ± 0.75	38.92 ± 0.85	0.836	0.960
Newborn weight (g)	3338.99 ± 327.26	3317.81 ± 333.14	3314.18 ± 323.72	3323.50 ± 337.73	0.367	0.636
Fetal sex, Male (%)	201 (53.3)	212 (50.6)	120 (46.9)	92 (56.4)	0.443	0.120
Apgar score at 1 min	10 (10, 10)	10 (10, 10)	10 (10, 10)	10 (10, 10)	0.361	0.539
Apgar score at 5 min	10 (10, 10)	10 (10, 10)	10 (10, 10)	10 (10, 10)	0.244	0.275

Data are given as mean ± SD, n (%) or median (interquartile range, IQR)

^a^ The comparison between ECC group and DCC group

^b^ The comparison among ECC group and the two subgroups of DCC group

**Table 2:** Neonatal transcutaneous bilirubin levels and the rate of phototherapy

	ECC group (*n* = 377)	DCC group (*n* = 419)	DCC (30–60 s) group (*n* = 256)	DCC (61–120 s) group (*n* = 163)	*p*^a^	*p*^b^
Cord clamping time (s)	–	60 (50, 70)	53.5 (45, 60)	76 (70, 86)	–	–
Transcutaneous bilirubin in day 0 of age (mg/dl) (< 6 mg/dl)	1.49 ± 0.90	1.70 ± 0.78 *	1.73 ± 0.80 *	1.66 ± 0.74	0.004	0.012
Transcutaneous bilirubin in day 1 of age (mg/dl) (< 6 mg/dl)	4.88 ± 1.50	4.78 ± 1.32	4.82 ± 1.37	4.72 ± 1.24	0.335	0.499
Transcutaneous bilirubin in day 2 of age (mg/dl) (< 9 mg/dl)	8.57 ± 1.89	8.53 ± 1.75	8.51 ± 1.78	8.57 ± 1.71	0.799	0.928
Transcutaneous bilirubin in day 3 of age (mg/dl) (< 12 mg/dl)	10.96 ± 2.19	10.96 ± 2.06	11.00 ± 2.08	10.91 ± 2.04	0.995	0.912
Transcutaneous bilirubin in day 4 of age (mg/dl) (< 15 mg/dl)	11.80 ± 2.17	11.74 ± 2.29	11.77 ± 2.33	11.70 ± 2 .23	0.700	0.876
Transcutaneous bilirubin in day 5 of age (mg/dl) (< 15 mg/dl)	11.73 ± 2.12	11.91 ± 2.58	11.96 ± 2.69	11.85 ± 2.44	0.357	0.606
neonates needing phototherapy (%)	86 (22.8)	91 (21.7)	54 (21.1)	37 (22.7)	0.711	0.867

Data are given as mean ± SD, n (%) or median (IQR)

^a^ The comparison between ECC group and DCC group

^b^ The comparison among ECC group and the two subgroups of DCC group

^*^ A statistically significant difference comparing with ECC group (one-way ANOVA with LSD post hoc test): *p* < 0.05

The hematological status of neonates on day 1, 2 and 3 was shown in Table 3. In comparison with the ECC group, delayed umbilical cord clamping slightly enhanced the hemoglobin and hematocrit levels on the first two days, but the differences were not statistically significant (*p* > 0.05). On the third day, the hemoglobin level in the DCC group (*n* = 32; 195.66 ± 22.95 g/L) was significantly higher than that in the ECC group (*n* = 33; 183.48 ± 22.03 g/L) (*p* < 0.05). The hemoglobin in the 30–60 s DCC group was 199.35 ± 24.82 g/L, and in the 61–120 s DCC group was 191.47 ± 20.67 g/L. However, when the two subgroups of DCC were compared with the ECC group respectively, the *p*-value was slightly greater than 0.05, which might be due to the small sample size. And there was no significant difference in hemoglobin between the two subgroups. Likewise, the higher hematocrit level on day 3 in the DCC group, but the difference did not reach statistical significance (*p* = 0.052). Increasing the duration of delayed cord clamping from 60 s to 120 s was not associated with further increases in hemoglobin and hematocrit levels of neonates on the third day after birth. Additionally, no neonates were diagnosed with polycythemia on day 3 in the ECC group, while 3 neonates (17.7%) had the polycythemia in the 30–60 s DCC group, and there was a statistically significant difference in the rate of neonatal polycythemia between the ECC group and two DCC subgroups (*p* < 0.05).

**Table 3:** The hematological status of neonates within 3 days of life

	ECC group	DCC group	DCC (30–60 s) group	DCC (61–120 s) group	*p*^a^	*p*^b^
Day 1	(*n* = 61)	(*n* = 53)	(*n* = 31)	(*n* = 22)		
Neonatal hemoglobin (g/L)	200.85 ± 22.33	201.58 ± 24.58	198.94 ± 25.73	205.32 ± 22.91	0.868	0.613
Neonatal hematocrit (%)	62.44 ± 7.87	63.07 ± 8.71	61.78 ± 9.01	64.89 ± 8.11	0.686	0.371
Neonatal polycythemia (%)	8 (13.1)	11 (20.8)	4 (12.9)	7 (31.8)	0.275	0.105
Day 2	(*n* = 25)	(*n* = 46)	(*n* = 27)	(*n* = 19)		
Neonatal hemoglobin (g/L)	203.32 ± 17.40	205.07 ± 21.52	204.96 ± 22.18	205.21 ± 21.14	0.729	0.941
Neonatal hematocrit (%)	62.92 ± 6.52	64.09 ± 7.84	63.78 ± 8.33	64.52 ± 7.28	0.530	0.778
Neonatal polycythemia (%)	4 (16.0)	11 (23.9)	5 (18.5)	6 (31.6)	0.435	0.417
Day 3	(*n* = 33)	(*n* = 32)	(*n* = 17)	(*n* = 15)		
Neonatal hemoglobin (g/L)	183.48 ± 22.03	195.66 ± 22.95 *	199.35 ± 24.82	191.47 ± 20.67	0.033	0.064
Neonatal hematocrit (%)	55.73 ± 6.55	59.58 ± 8.97	60.67 ± 10.15	58.35 ± 7.56	0.052	0.108
Neonatal polycythemia (%)	0 (0.0)	3 (9.4)	3 (17.6) *	0 (0.0)	0.114	0.026

Data are given as mean ± SD or n (%)

^a^ The comparison between ECC group and DCC group

^b^ The comparison among ECC group and the two subgroups of DCC group

^⁎^ A statistically significant difference comparing with ECC group (one way ANOVA with LSD post hoc test): *p* < 0.05

## Discussion

Professional guidelines on delayed cord clamping are constantly updated. The Enhanced Recovery After Surgery Society guideline recommended the timing of cord clamping in term newborns was at least 1 min [18]. The ACOG recommended at least 30–60 s in preterm and term neonates [2]. To date, the optimal timing of DCC in cesarean section remains uncertain. In our study, we defined DCC as a delay of cord clamping for at least 30 s, which was consistent with the ACOG guideline. We investigated the short-term effects of DCC at different time on neonatal jaundice, the rate of jaundice requiring phototherapy and the early hematological status of newborns.

In this trial, we found that clamping the umbilical cord beyond 30 s increased the transcutaneous bilirubin on the day of birth, while this effect disappeared from the first to the fifth day of birth. The transiently elevated bilirubin from the amount of extra blood volume on the day of birth might be fastly metabolized, which caused no damage to the newborns. Clamping the cord at 30–60 s significantly increased the rate of neonatal polycythemia without the enhanced requirement of neonatal phototherapy and other adverse outcomes.

ECC rather than DCC is often implemented in cesarean section due to the fear of increasing requirement of phototherapy for neonatal jaundice and excessive maternal blood loss. However, our study demonstrated that DCC did not increase the rate of phototherapy, although DCC resulted in elevated transcutaneous bilirubin on the day of birth temporarily and the increasing rate of polycythemia on the third day after birth. This outcome was consistent with previous studies [4, 7]. What’s more, the conclusion of the little relationship between DCC in vaginal delivery and the requirement of phototherapy was approved by most researches [3, 6, 19]. Conversely, our previous study [5] and the report from Japan [20] observed that DCC led to a higher risk of neonatal jaundice requiring phototherapy in healthy term newborns. The different findings may be due to the diversities in the study design, the sample size, and the study population. The difference between our previous study and the current study is due to different delivery methods. It was reported that cesarean section was associated with a less placental transfusion compared with vaginal delivery [15]. In addition, no significant difference in maternal postoperative hemorrhage was found between the ECC group and the DCC group [4, 21], which is corroborated our findings (the results not shown). Therefore, delayed cord clamping in healthy term neonates is a safe procedure during cesarean section without apparent harmful effects on the neonates and their mothers.

For term neonates, several randomized controlled trials [3, 6, 22, 23] had reported DCC resulted in improved hemoglobin levels at birth or within the three days of life, which was in agreement with our results. But few studies [3, 22, 23] took the effect of different delivery methods on DCC into account. The pattern of placental transfusion differed between vaginal delivery and cesarean section delivery [15]. Our study demonstrated delayed clamping at 30–60 s (the median time 53.5 s) increased hemoglobin and hematocrit levels on the third day after birth. Increasing the duration of cord clamping from 60 s to 120 s (the median time 76 s) did not result in further increases in hemoglobin and hematocrit levels but led to a decreasing trend, which may be the result of placental blood flow reflux. Clamping the cord at 30–60 s in cesarean section may be a better choice rather than 61–120 s.

However, a randomized controlled trial in 2019 showed that the hematocrit in a capillary at day 2 of life increased by 6% in neonates receiving DCC beyond 60 s during the cesarean section [7]. The conflicting results suggested that hematocrit and hemoglobin levels within several days could not completely reflect the impact of DCC at different time on placental transfusion. Multicenter large sample studies with long-term follow-up for the neonate hemoglobin levels are required for more reliable data.

The short-term beneficial effects of DCC in hemoglobin and hematocrit could make sense in the growth and development of newborns. A prospective study showed that the difference in cognitive function could not be eliminated between healthy formerly iron-deficient anemic children and normal ones after iron treatment for ten years [10]. Several studies confirmed that DCC improved iron status at 2 [9], 4 [24] and 6 [25] months of age in term neonates. In the 4th month, the level of ferritin in neonates born by elective cesarean section with DCC at 30 s was higher than those born vaginally with ECC and similar to those born vaginally with DCC at 180 s [26]. Reportedly, effective placental transfusion merely occurred in the first 40 s after birth in cesarean section [14]. A similar result was found in our study that DCC at 61–120 s could not further improve hematological status compared with DCC at 30–60 s. DCC at 30–60 s should be an optimal time in cesarean section, which could benefit the neonates in the long term.

Limitations of this study include the small blood sample size of the DCC group on day 3 for the ethical reason that heel peripheral blood collection is an invasive procedure, and focusing on the effects of DCC in cesarean section on short-term hematological status rather than long-term hematological effects. The strength of this study was repeated measurement in bilirubin level and blood indicators, ensuring the accuracy of the effect of DCC on neonates. Additionally, our trial was one of the few researches on the implementation of DCC during cesarean section.

## Conclusions

In cesarean section, a delay in cord clamping for at least 30 s improved the hematological status of term neonates on day 3 of life without the enhanced requirement of phototherapy for neonatal jaundice. Delayed cord clamping beyond 60 s did not further improve hematological status. Delayed cord clamping at 30–60 s is a simple, economical, effective and safe procedure that can be recommended in term cesarean section.

## Data Availability

The datasets used and/or analyzed during the current study are available from the corresponding author on reasonable request.

## Abbreviations

ACOG: American College of Obstetricians and Gynecologists
DCC: Delayed cord clamping
ECC: Early cord clamping
SD: Standard deviation
IQR: Interquartile range
